# Genome Sequence of *Listeria monocytogenes* Plasmid pLM-C-273 Carrying Genes Related to Stress Resistance

**DOI:** 10.1128/genomeA.01125-16

**Published:** 2016-10-13

**Authors:** Lindsay Liang, Saravanamuttu Gnaneshan, Rafael A. Garduño, Gustavo V. Mallo

**Affiliations:** a Public Health Ontario, Toronto, Canada; b Department of Laboratory Medicine and Pathobiology, University of Toronto, Toronto, Canada; c Canadian Food Inspection Agency, Dartmouth Laboratory, Dartmouth, Canada

## Abstract

Mobile genetic elements in bacteria, such as plasmids, act as important vectors for the transfer of antibiotic resistance, virulence, and metal resistance genes. Here, we report the genome sequence of a new plasmid pLM-C-273, identified in a *Listeria monocytogenes* strain isolated from a clinical sample in Ontario, Canada.

## GENOME ANNOUNCEMENT

*Listeria monocytogenes* is highly resistant to environmental stresses that usually limit bacterial growth, such as low temperatures, fluctuations in pH, high salt concentrations, and metal ions (1), making its eradication from food manufacturing plants difficult. In general, plasmids have been implicated as genetic vectors in the interbacterial transfer of antibiotic resistance (2), virulence factors (3), and metal ion resistance factors (4). Here, we report the nucleotide sequence of *L. monocytogenes* plasmid pLM-C-273, which was derived from the whole-genome sequence of the strain LM-C-273. *L. monocytogenes* strain LM-C-273 is a clinical isolate recovered from a sample obtained in 2009 in Ontario, Canada, and belongs to multilocus sequence type 5 (ST5) (5).

Short-read sequences for LM-C-273 were obtained using the Illumina HiSeq 2500 technology (Illumina, San Diego, CA), and read quality was assessed using FastQC 0.1 (http://www.bioinformatics.babraham.ac.uk/projects/fastqc/). These short reads were then assembled using plasmidSpades (6), which uses the read coverage of generated contigs to distinguish between chromosomal and plasmid DNA. The assembly of plasmid contigs rendered a sequence that was 99,874 nucleotides in length in eight scaffolds, with a G+C content of 36.0%. The assembly was annotated with tools from the Victorian Bioinformatics Consortium-Prokka, using its default parameters (7). A total of 123 predicted coding sequences (CDSs) were annotated.

When the nucleotide sequence for pLM-C-273 was compared to the nonredundant nucleotide sequences database of the National Center for Biotechnology Information (NCBI), it was found that 59% of pLM-C-273’s sequence matched *L. monocytogenes* strain N1-011A plasmid (accession no. NC_022045.1) with 99% identity. Analysis of the predicted gene products encoded in the remaining 41% of pLM-C-273’s sequence that was not homologous to *L. monocytogenes* strain N1-011A plasmid, was done with PHAge Search Tool (PHAST) (8) and revealed several phage-related proteins. Specifically, these phage proteins seem to have originated from *Listeria* phage A118 (accession no. NC_003216.1), *Listeria* phage vB LmoS 188 (accession no. NC_028871.1), and *Listeria* phage A006 (accession no. NC_009815.1), suggesting a promiscuous origin for pLM-C-273. Further examination of the annotated genes in pLM-C-273 revealed a PemK interferase sequence (9), an NADH peroxidase protein reported to be responsible for hydrogen peroxide detoxification (10), a copper-transporting P-type ATPase, and a multicopper oxidase implied in copper detoxification (11), as well as a cadmium resistance operon (*cadA* and *cadC*) (12). Collectively, these findings suggest the involvement of pLM-C-273 in providing fitness advantages to strain LM-C-273, particularly related to resistance against metal ions and oxygen radicals. Analysis of *L. monocytogenes* plasmids can help better understand the clinical emergence of subgroups of *L. monocytogenes* strains with the potential to adapt more efficiently to disinfection protocols in food manufacturing plants, or to survive the food preparation process, as the results of horizontal gene transfer events.

### Accession number(s).

The genome sequence of pLM-C-273 plasmid has been deposited in the GenBank database under the accession numbers KX467250 to KX467259.
